# Development of a Colloidal Gold Immunochromatographic Assay Strip Using a Monoclonal Antibody for the Rapid Detection of Ofloxacin

**DOI:** 10.3390/foods13244137

**Published:** 2024-12-20

**Authors:** Xiaolan Li, Jin Huang, Na Li, Mahmoud Salah, Shuoning Guan, Wenwen Pan, Ziyi Wang, Xinghua Zhou, Yun Wang

**Affiliations:** 1 School of Food and Biological Engineering, Jiangsu University, Zhenjiang 212013, China; 2222318040@stmail.ujs.edu.cn (X.L.); 3220910011@stmail.ujs.edu.cn (J.H.);; 2 Department of Environmental Agricultural Science, Faculty of Graduate Studies and Environmental Research, Ain Shams University, Cairo 11566, Egypt

**Keywords:** ofloxacin, monoclonal antibody, immunochromatographic strip, rapid detection

## Abstract

The livestock industry uses ofloxacin, an antibiotic, to prevent several animal diseases; however, the overdose of ofloxacin used in animal farming treatments may appear in food products and cause some adverse human health effects. Hence, there is an immediate need to develop a method suitable for on site large-scale detection of ofloxacin residues in animal-derived foods. This study aimed to prepare a monoclonal antibody with high sensitivity and affinity for ofloxacin by re-synthesizing the ofloxacin hapten and synthesizing the corresponding complete antigen. The IC_50_ of the enzyme-linked immunosorbent assay (ic-ELISA) was 0.13 ng/mL, and the detection limit was 0.033 ng/mL. The visual detection limit of the established colloidal gold immunochromatographic test strip, for the visual detection of actual samples, was 1 ng/g. In summary, this work establishes a rapid detection method of ofloxacin residues on the basis of colloidal gold immunochromatography that is suitable for actual detection.

## 1. Introduction

**Figure 1:**
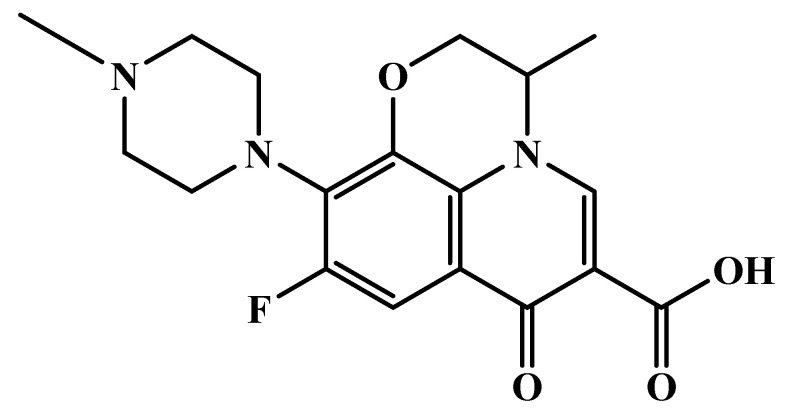
Chemical structure of OFL.

Ofloxacin (OFL, see Figure 1) is a quinolone antibacterial drug with broad-spectrum antimicrobial effects and is, therefore, often used to treat respiratory tract infections, genitourinary infections, and infections caused by sensitive bacteria [1]. It is an important antibiotic for domestic animals [2]. However, with its widespread use, the problems caused by its residues have attracted more and more attention. The long-term low intake of animal-derived food and water containing residues of OFL by humans will lead to the development of drug resistance in humans in addition to potential health hazards, such as toxic effects (central nervous system toxicity), peripheral neuropathy, allergic reactions, etc. [3]. In 2015, China issued the Ministry of Agriculture Announcement No. 2292, stating that food should not contain OFL due to the increasing safety risk of such residues. Despite this, cases of ofloxacin residues exceeding the sampling limit still occur. Hence, there is an immediate need to develop a method suitable for on site large-scale detection of ofloxacin residues [4].

The common methods for detecting ofloxacin residues include instrument detection and immunoassay. The instrument detection method includes HPLC [5,6,7,8], HPLC-MS/MS [9,10,11,12], and surface-enhanced Raman spectroscopy (SERS) [13]. Although the instrumental detection method is characterized by high sensitivity and accuracy, its high cost and complicated operational procedures render it unsuitable for on site detection. The high sensitivity and simple operation of immunoassay lead to its widespread use in detecting antibiotic residues. Huet et al. [14] established a dc-ELISA universal detection method for 15 FQs antibiotics in which the estimated detection limit of OFL was 25 μg/kg. Zhang et al. [15] developed an ic-ELISA method to detect 13 FQs drugs synchronously and the detection limit for OFL was set at 1.15 μg/L. This study primarily employs colloidal gold immunochromatography, a method known for its simple operation, low detection cost, and suitability for rapid screening in the field [16]. Yuping Wang [17] developed a colloidal gold immunochromatographic assay to detect several fluoroquinolones. The detection limits (LODs) for OFX and MBF in milk were 3.5–8.9 ng/mL. Additionally, Byzova et al. [1] developed a competitive colloidal gold immunochromatographic assay for detecting OFL residues with a detection limit of 30 ng/mL.

In this work, monoclonal antibodies with high sensitivity and high affinity for ofloxacin were prepared. On this basis, the ic-ELISA method and the colloidal gold immune test strip method were established. The IC_50_ of ic-ELISA was 0.13 ng/mL, and the detection limit was 0.033 ng/mL. The visual detection line of the colloidal gold test strip was 1 ng/g, which met the national requirements and was lower than that reported in the previous literature.

## 2. Materials and Methods

### 2.1. Reagents and Equipment

Ovalbumin (OVA), bovine serum albumin (BSA), horseradish peroxidase-labeled goat anti-mouse IgG, 4,4′-Bi-2,6-xylidine, Carbodiimides (EDC), Freund’s incomplete adjuvant, Freund’s complete adjuvant, Citric acid trisodium salt, Chloroaquinic acid (HAuCl_3_·4H_2_O) were purchased from Sigma (St. Louis, MO, USA). Fetal bovine serum (FBS), trisodium citrate and thymidine medium, hypoxanthine–thymidine medium, and RPMI-1640 medium were purchased from GIBCO (Carlsbad, CA, USA). All other reagents were acquired from the National Pharmaceutical Group Chemical Reagent Co., Ltd. (Shanghai, China). The microplate reader (HF4500) was purchased from Huaan Maike Biotechnology Co., Ltd. (Beijing, China). The CT300 CNC strip cutting machine and HM3030 XYZ three-dimensional dispensing platform were obtained from Shanghai Kinbio Tech Co., Ltd. (Shanghai, China). Ethics and statement: animals (6- to 8-week-old female BALB/c mice) for the experiments were obtained from Kavans Laboratory Animal Co., Ltd., Changzhou, China (Certificate No. 202122406).

### 2.2. Modification of Ofloxacin Semi-Antigen

**Figure 2:**
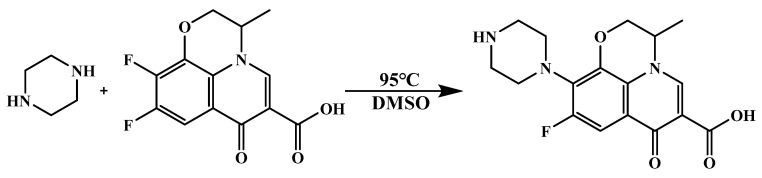
Modification of the hapten of OFL.

To improve the sensitivity of the assay, a new semi-antigen (Figure 2) was formed by resynthesizing the ofloxacin—which lacks a methyl group compared with the original ofloxacin—and using this to synthesize the corresponding envelope, thus improving the sensitivity of the assay with the advantage of the heterologous envelope [18]. The method of modifying the OFL hapten structure is as follows [19]:

A total of 172 mg of 1,4-diazacyclohexane was weighed and added to a solution of DMSO (3 mL) containing 281 mg of 9,10-difluoro-2,3-dihydro-3-methyl-7-oxo-7H-pyrido[1,2,3-DE]-1,4-benzoxazine-6-carboxylic acid. The suspension was stirred at 95 °C for 12 h. Then, 10 mL of acetone was added to the mixture. The brown residue obtained was ground in acetone (10 mL) to give the expected compound as an off-white powder.

### 2.3. Synthesis of Complete Antigens

**Figure 3:**
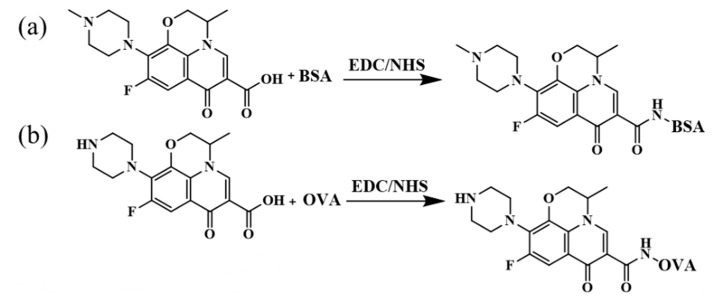
Preparation of immunogen and coating antigen ((**a**) OFL immunogen; (**b**) OFL coating antigen).

The specific steps for the preparation of complete antigen (as shown in Figure 3) by the activated ester method [20] are as follows: the immunogen was prepared by coupling the unmodified OFL with BSA, and the coating antigen was prepared by coupling the modified OFL with OVA. Amounts of 6.4 mg of semi-antigen, 4.1 mg of N-hydroxysuccinimide (NHS), and 6.8 mg of 1-ethylcarbodiimide hydrochloride (EDC) were weighed and dissolved in 0.6 mL of dimethylformamide (DMF) and stirred under room-temperature conditions overnight to achieve activation. The activated semi-antigen solution was dropped into a carbonate buffer (10 mL) containing 25.5 mg BSA or 20 mg OVA, stirred at 25 °C, and reacted overnight. Afterward, dialysis was performed in phosphate buffer at 4 °C for 72 h, and the dialysate was replaced every 8 h. After dialysis, the samples were stored at −20 °C for later use.

### 2.4. Preparation of Monoclonal Antibodies

The initial immunization was performed with an immunogen dose of 100 µg diluted in 0.9% NaCl solution and fully mixed with an equal volume of Freund’s complete adjuvant. Then, one booster immunization was performed every two weeks at a dose of 50 ug, again diluted with 0.9% NaCl solution, and fully mixed with the equal volume of Freund’s incomplete adjuvant. Starting from the third immunization, ic-ELISA analyzed the sera of each group of mice.

The titer and inhibition rate of the sera are generally the two main considerations in the screening process of mice, in which the titer represents the affinity of the prepared antibody; the inhibition rate is the main indicator of the specific recognition of the antibody, and its calculation formula is as follows:

$$Inhibition\;rate=\left(1-\frac{B}{B_{0}}\right)\times 100\%$$

where B_0_ is the absorbance value of the negative control group; and B is the absorbance value of the detection group.

For better cell fusion, the mouse spleen cells with the highest titer and inhibition rate were mixed with Sp 2/0 myeloma cells at a ratio of 1:7 and fused with 1 mL of PEG 1500 [21,22]. The fused cells were cultured with the HAT medium for 8 days and then replaced with the HT medium. Ic-ELISA screened positive wells.

The hybridoma cells with the highest titer and the best sensitivity were injected intraperitoneally into mice using a limited dilution method with 3 subcloning, and then the mouse ascites were collected after one to two weeks. The purification method of the ascites was ammonium sulfate precipitation, and the purified monoclonal antibody was stored at −20 °C.

### 2.5. Determination of Antibody Affinity, Sensitivity, and Specificity

The type of monoclonal antibody was detected by the kit and the affinity constant (Ka) was determined by ic-ELISA [23,24]. The formula for calculating Ka is as follows:

$$Ka=\frac{n-1}{2(n\left[Ab\right]t-\left[Ab^{\prime }\right]t)}$$

where n = [Ag]t/[Ag′]t, wherein “[Ag]t” and “[Ag′]t” are two different coating antigens concentrations, and “[Ab]t” and “[Ab′]t” are the concentration of OFL-mAb at 50% OD_450_ nm at these two coating antigens concentrations.

The sensitivity of the antibody is usually expressed by half inhibitory concentration (IC_50_). The specificity was expressed as the cross-reactivity (CR), and the cross-reactivity was calculated according to the following formula:

$$CR\left(\%\right)=\left(\frac{{IC}_{50}\;of\;OFL}{{IC}_{50}\;of\;analogs}\right)\times 100\%$$

### 2.6. Selection of the ic-ELISA Working Conditions

In the process of ic-ELISA, there are many factors affecting it. Therefore, to obtain the best detection results, the choice of containment conditions (37 °C containment for 1 h, 37 °C containment for 2 h, 4 °C overnight containment, 4 °C containment for 1 h, 4 °C containment for 2 h), competition time (0.125 h, 0.25 h, 0.5 h, 1 h), standard dilution pH (5, 6, 7.4, 8.6, 9.6), and ionic strength (0%, 0.5%, 1%, 1.5%, 2%) were optimized. Then, standard curves were established under these different conditions. IC_50_ and A_max_ (absorbance at zero concentration at 450 nm) were used to evaluate the performance of mAb in ic-ELISA [25,26,27,28,29,30].

### 2.7. Preparation of Colloidal Gold-Labeled Antibody

According to the previous research literature, a sodium citrate reduction method was used for the preparation of colloidal gold [31,32], described as follows: Take 100 mL of 1% aqueous chloroauric acid in a flask and heat it until boiling, then add 4 mL of 1% trisodium citrate while stirring. In this process, when the reaction color of the solution changes to burgundy, the heating is stopped, allowing the solution to cool down to 20–25 °C and maintaining it at 4 °C.

Afterward, the pH of the colloidal gold solution was adjusted to 8.5 with 0.1 M K_2_CO_3_ solution; then, 6 uL of antibody was added to each milliliter of colloidal gold and stirred in the solution for 45 min. Then, 100 uL of 10% bovine serum albumin solution was added to each milliliter and stirring was continued for 2 h at 20–25 °C, followed by centrifugation at 8000 rpm for 15 min. Then, the precipitate was washed the obtained. Soluble impurities were removed by centrifugation, and the washing steps were repeated twice. Finally, the precipitate was resuspended and stored at 4 °C in a dark place.

### 2.8. Preparation of Colloidal Gold Immunochromatographic Assay Strips

**Figure 4:**
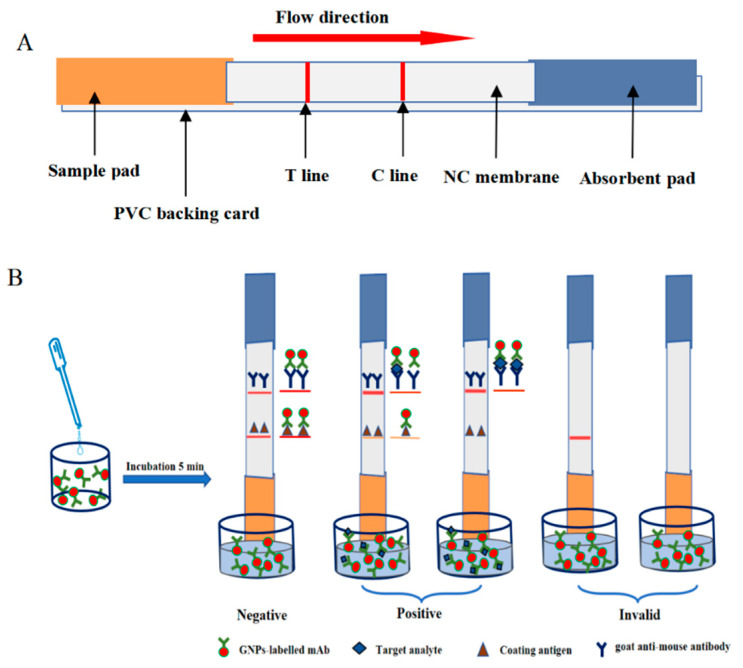
The basic principle of the colloidal gold immunochromatographic assay: (**A**) structure and (**B**) principle.

The immunochromatographic strip mainly consists of a sample pad, a backing plate (PVC), a nitrocellulose membrane (NC membrane), and an absorbent pad, as shown in Figure 4A. The coating antigens formed the detection line (T line), originally immobilized on the NC membrane, and the quality control line (C line) was formed by the sheep anti-mouse antibody (secondary antibody), immobilized on the NC membrane.

### 2.9. Principle of the Colloidal Gold Immunochromatographic Assay Strips

Figure 4B demonstrates the fundamental mechanism of a colloidal gold immunochromatographic test strip. The test strip is inserted into a microwell and the sample, mixed with gold-labeled antibody, migrates from the sample pad toward the absorbent end. Initially, the gold-labeled antibody binds to the T line, while the unbound portion continues to attach to the secondary antibody at the C line. In the case of a negative sample, the test strip exhibits two red bands of comparable intensity, or the T line may appear slightly darker than the C line. Conversely, if the sample is positive, the gold-labeled antibody preferentially binds to the target substance in the sample, resulting in reduced binding to the T line, and the intensity of the T line is weakened. The point where the T line starts to brighten signifies the visual limit of detection (vLOD) of the test strip.

### 2.10. Optimization of Working Conditions of Immunochromatographic Strip

The amount of antibody and the pH of the working environment during the preparation of gold-labeled antibodies are two key factors that will affect the levels of detection.

#### 2.10.1. Optimization of the Gold-Labeled Antibody pH

A total of 1 mL colloidal gold solution was added to the centrifuge tube, and then, 0, 2, 4, 6, 8, 10, 12, 14, and 16 μL 0.1 M K_2_CO_3_ solution was added, respectively. After mixing, 10 uL 0.5 mg/mL monoclonal antibody was added. Then, after 30 min the color of the mixed solution was observed. The group with unchanged color and the least amount of K_2_CO_3_ solution was selected as the best dosage.

#### 2.10.2. Optimization of Gold-Labeled Antibody Concentration

Based on the optimal K_2_CO_3_ dosage, antibodies of 4, 6, 8, 10, and 12 μg were added to the centrifuge tubes with 1 mL of colloidal gold solution. After standing at 25 °C for 5 min, the group that had an unchanged color and the smallest number of antibodies displayed the optimal antibody concentration.

#### 2.10.3. Optimization of the Concentration of Coating Antigen in T Line

The coating antigens with concentrations of 0.2, 0.5, and 1 ng/mL were coated on the T line using an instrument, and the color reaction was carried out after drying. The group with the most stable color rendering and the lowest visual detection limit was used as the optimal coating concentration.

### 2.11. The Actual Sample Detection Experiment

The actual samples included pork, fish, and chicken. Amounts of 0, 1, 2, and 4 ng/g were used as the added concentration of the OFL standard for standard addition detection, and the visual detection line of the colloidal gold immunochromatographic test strip was judged through megascopic examination.

## 3. Results and Discussion

### 3.1. Identification of Ofloxacin Semi-Antigen

**Figure 5:**
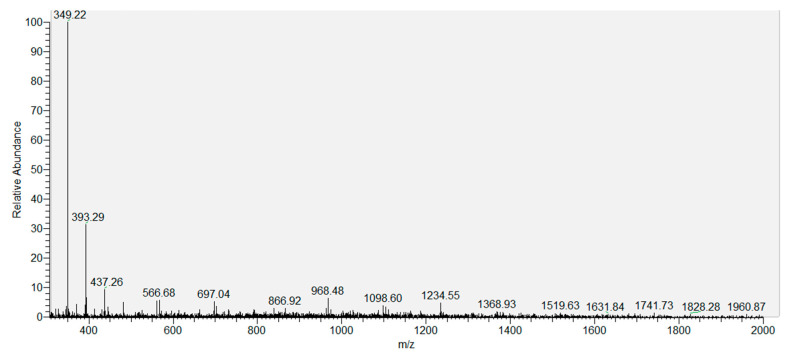
Mass spectrometry of the modified OFL.

**Figure 6:**
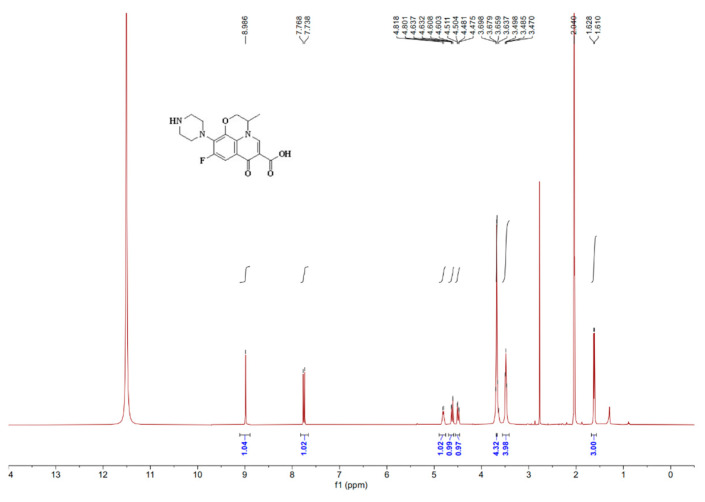
The ^1^H NMR spectrum for the modified OFL.

Figure 5 and Figure 6 show the mass spectra and spectroscopy of the modified OFL semi-antigen. There is a molecular ion peak of 349.22 m/z, which corresponds to the molecular weight of the modified OFL (347.33); therefore, the semi-antigen was successfully modified.

### 3.2. Identification of Complete Antigen

#### 3.2.1. UV Characterization of OFL Complete Antigen

**Figure 7:**
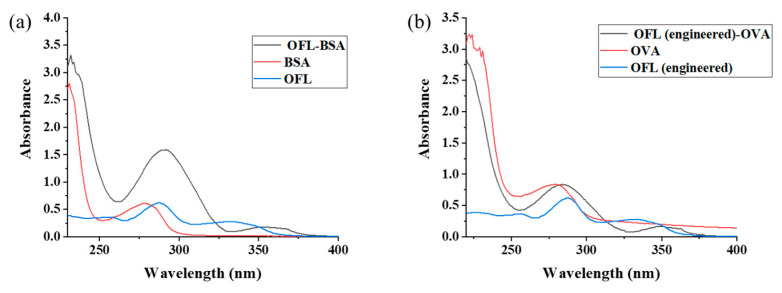
UV–vis spectrum of OFL immunogen (**a**) and coating antigen (**b**).

As shown in Figure 7a,b, OFL has a characteristic absorption peak between 275 and 285 nm, and the maximum absorption peaks of OFL-BSA and OFL-OVA are at 285 nm and 280 nm, respectively, which are shifted compared with those of the carrier proteins. This indicates that the coupling of the OFL immunogen and encapsulant was successful [20].

#### 3.2.2. Determination of Complete Antigen Concentration

**Figure 8:**
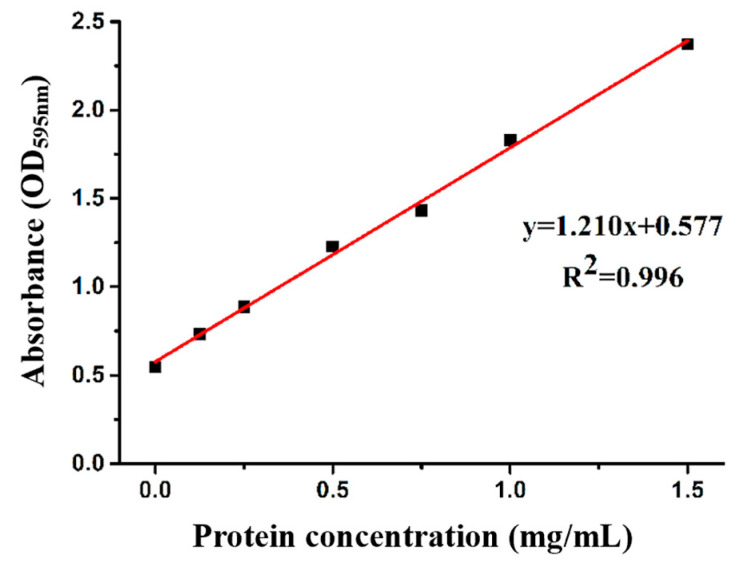
Protein standard curve.

The measurements of immunization, antibody potency, and inhibition rate are based on the concentrations of the immunogen and encapsulant. Figure 8 shows the established protein standard curve; the concentration of the immunogen (OFL-BSA) is 5.23 mg/mL according to the standard curve, and the concentration of the coating antigen (OFL (engineered)-OVA) is 5.25 mg/mL.

### 3.3. Selection of Hybridoma Cells

Three BALB/c mice of the same batch with similar body weights were selected and immunized with the prepared immunogen five times, and the anti-serum was detected by ic-ELISA. The serum test results obtained by the last immunization are shown in Table 1. The coating antigen concentration of 0.3 μg / mL and the anti-serum dilution of 3000 times were selected for serum detection. After detection, the effect of the heterologous coating antigen was better than that of the homologous coating antigen; therefore, the heterologous coating antigen was used for the following series of experiments. In three mice, the titer and inhibition rate of No. 3 mice were not as good as those of No. 1 and No. 2; therefore, No. 3 mice were not considered. In contrast, the inhibition of No. 1 and No. 2 mice was not much different but the titer of No. 1 was slightly higher than that of No. 2. Therefore, the No. 1 mice were selected for the subsequent experiments.

**Figure 9:**
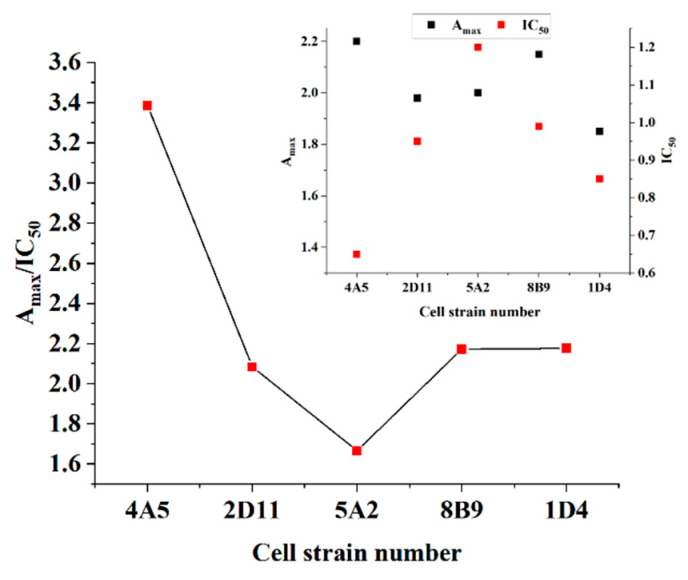
The screening results of hybridoma cells of OFL.

After the fusion of Sp 2/0 cells and B cells, the detected positive cells need to be sub-cloned three times using a limited dilution method to screen out the hybridoma cell lines with high purity and meet the requirements. After subcloning screening, five OFL hybridoma cell lines were successfully obtained. The results are shown in Figure 9 (The detailed data are presented in Supplementary Materials). According to the comprehensive data, the titers of antibodies produced by cell line 4A5 and cell line 8B9 were not much different but the IC_50_ and A_max_/IC_50_ values of 4A5 were better than those of 8B9. Therefore, selecting cell line 4A5 prepared the OFL monoclonal antibody and the establishment of related detection methods.

### 3.4. Identification of mAb

**Figure 10:**
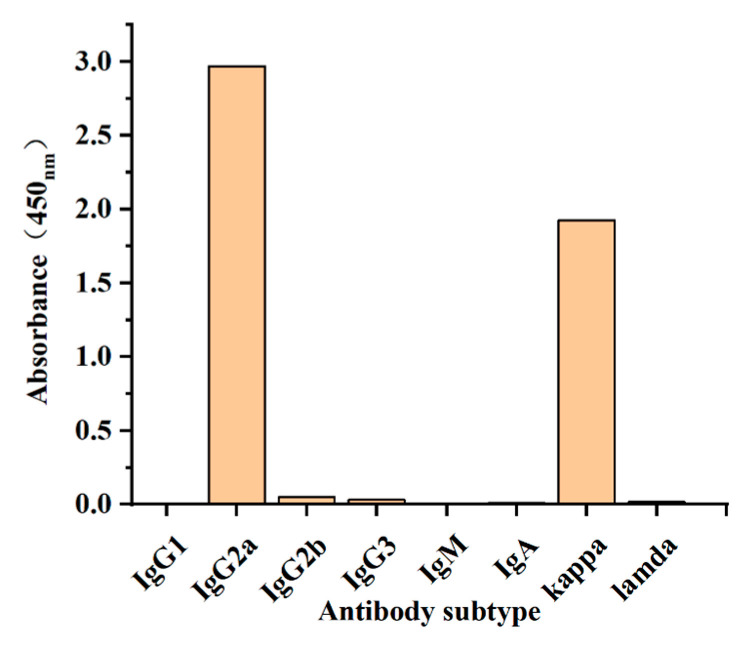
Subtype determination of mAb.

**Figure 11:**
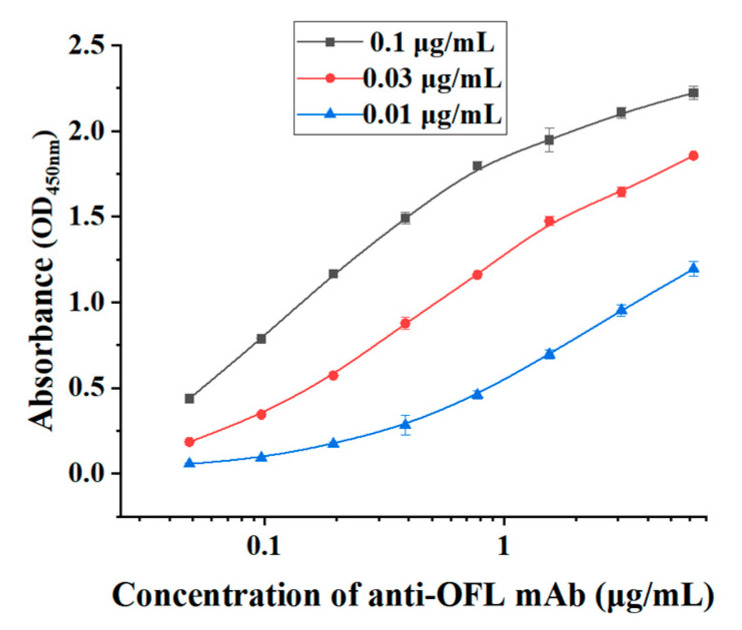
Affinity constant result of OFL antibody.

The subtype of monoclonal antibody was measured by the kit C060101-L (from Luoyang Baiaotong Experimental Material Center, Luoyang, China). As shown in Figure 10, the heavy chain subtype of OFL antibody was IgG2a, and the light chain subtype was kappa. The affinity constants of mAb were determined by selecting three coating antigen concentrations of 0.1, 0.03, and 0.01 μg/mL. Figure 11 displays the result of the affinity constant of the OFL antibody. According to the curve regression equation, the concentration of monoclonal antibody corresponding to OD_max/2_ was obtained. According to the affinity constant formula, Ka_1_ = 7.73 × 10^9^ L/mol, Ka_2_ = 1.32 × 10^10^ L/mol, and Ka_3_ = 8.63 × 10^9^ L/mol were calculated. The affinity constant of the OFL antibody is Ka = (Ka_1_ + Ka_2_ + Ka_3_) / 3 = 9.85 × 10^9^ L/mol. The results showed that the prepared OFL antibody had a high affinity.

### 3.5. Optimization Results of ic-ELISA Working Conditions

The optimal working point of the ic-ELISA method was obtained by the chessboard method, that is, the concentration of coating antigen was 0.03 μg / mL, and the concentration of antibody was 0.03 μg/mL.

**Figure 12:**
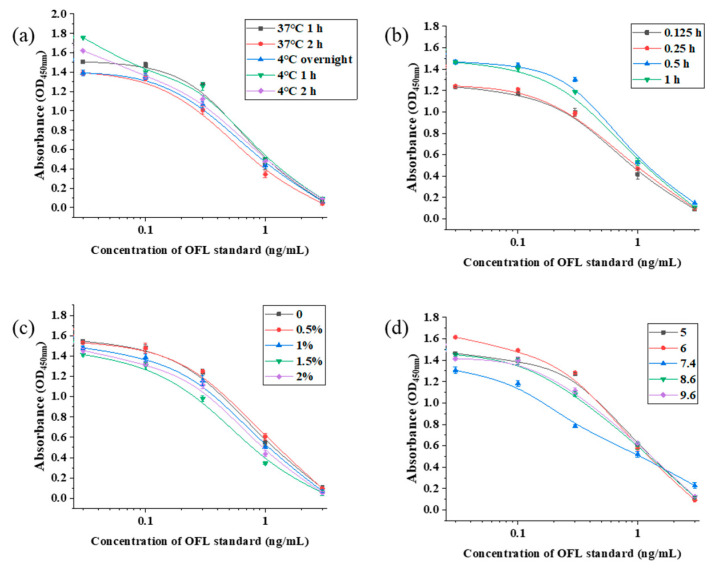
The optimization results of ELISA working conditions: blocking conditions (**a**), competition time (**b**), NaCl content (**c**), and pH (**d**).

Based on the optimal working point, the optimization of the OFL antibody ic-ELISA method is shown in Figure 12 and the specific data are shown in Table 2 as well as Table 3. The optimal containment conditions for OFL’s ic-ELISA method were 2 h of containment at 37 °C, a best competitive reaction time of 0.5 h, an optimal NaCl content of 1.5% for the standard dilutions, as well as an optimal pH of 7.4.

### 3.6. Establishment of Standard Curve

**Figure 13:**
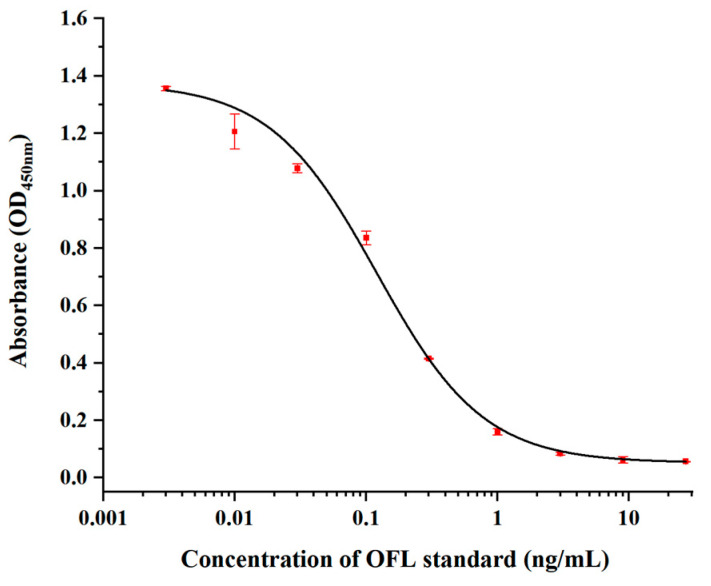
The standard inhibition curves of OFL.

In this work, IC_20_~IC_80_ is defined as the linear range of detection, and IC_20_ is defined as the minimum detection limit of the ic-ELISA. The ic-ELISA standard inhibition curve of OFL is shown in Figure 13. The corresponding equation is $y=-0.0086+1.303/[1+{\left(\frac{x}{0.13}\right)}^{1.01}]$, R^2^ = 0.994. The IC_50_ is 0.13 ng/mL, the detection limit is 0.033 ng/mL, and the linear detection range is 0.033~0.51 ng/mL.

### 3.7. Determination of Antibody Sensitivity and Specificity

Table 4 shows the cross-reactivities of the OFL monoclonal antibody with five other common structural analogs, among which the cross-reactivities with four structural analogs—lomefloxacin (LOM), pefloxacin (PEF), ciprofloxacin (CIP), and norfloxacin (NOR)—was low at less than 0.1% but the cross-reactivity with marbofloxacin (MBF) was higher, reaching 86.67%; therefore, the immunological assay established with this monoclonal antibody can make a preliminary judgment of OFL.

### 3.8. Identification of Colloidal Gold-Labeled mAb

**Figure 14:**
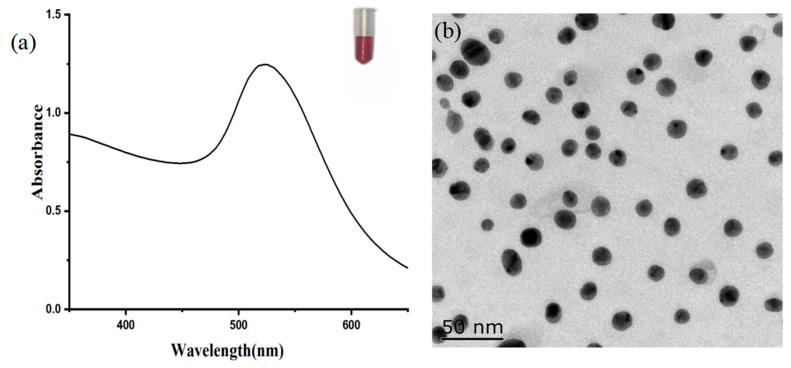
The characteristic images of colloidal gold particles: (**a**) UV–visible spectra, and (**b**) transmission electron microscopy images.

As shown in Figure 14, the color of the prepared colloidal gold solution was burgundy, clarified, and translucent without impurities, while the UV scanning graph showed that the prepared colloidal gold nanoparticles had the maximum absorbance value at 522 nm and the peak width was narrow.

### 3.9. Optimization of Parameters Related to Test Strips

The color of the gold-labeled antibody will change due to different pH values, and the naked eye will observe the color change, the results are summarized in Table 5, which show that the optimal amount of K_2_CO_3_ solution for synthesizing OFL gold-labeled antibodies is 12 μL.

The antibody concentration also affects the color of the gold-labeled antibody solution, and the naked eye can observe the color change; the results are summarized in Table 6, which show that the optimal concentration of antibody for preparing gold-labeled antibodies is 8 μg/mL.

Table 7 displays the optimization results of the concentrations of coating antigen and antibody of the OFL colloidal gold test strip. When the concentration of coating antigen is 0.5 mg/mL, the color of the T line gradually deepens with the increase in the amount of antibody. When 12 μL of the gold-labeled antibody is added, the gray value (by ImageJ software (Version: 1.52)) of the T line reaches 1004, and the vLOD has reached stability at 1 ng/mL. Therefore, the best detection performance of the colloidal gold test strip was established with 0.5 mg/mL of coating concentration and 12 μL of the gold-labeled antibody.

### 3.10. The Actual Samples Detected by the ic-ELISA Method and Colloidal Gold Immunochromatographic Test Strip

This study investigated three types of meat: pork, chicken, and fish. Table 8 provides the determination results of the recovery rate of OFL by ic-ELISA; these show that the recovery rate of OFL in pork samples was 91.0%~103.2%, the recovery rate of OFL in fish was 82.0%~99.6%, and the recovery rate of OFL in chicken was 86.0%~104.4%. The recovery rates for all three samples fell within the range of 80%~120% and the average coefficient of variation was below 5%. These results indicate that the colloidal gold test strip method is feasible for detecting OFL residues applied to the actual detection of these three types of meat products.

**Figure 15:**
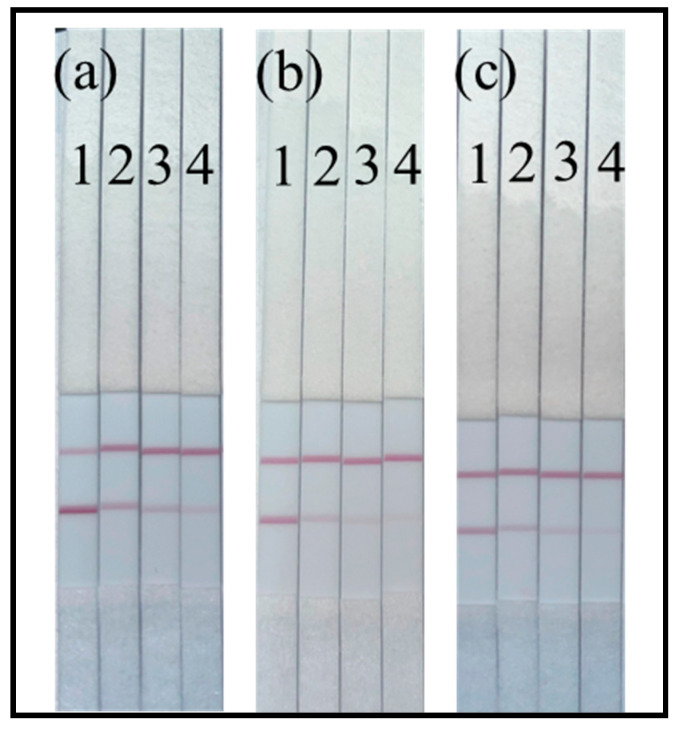
The images of colloidal gold immunochromatographic strip tests for OFL in (**a**) pork, (**b**) fish, and (**c**) chicken samples. (Note: the additional amounts of OFL standard in these samples are 1: 0 ng/g, 2: 1 ng/g, 3: 2 ng/g, 4: 4 ng/g).

The vLOD of the colloidal gold immunochromatographic test strip was determined. In samples with no OFL present, the T-line color would be similar to the C-line color. As the OFL concentration in the sample increased, the T-line color became lighter. The results of testing three meat samples using the colloidal gold immunochromatographic test strip method are shown in Figure 15. From the diagram, it can be observed that the vLOD of this method in the three actual samples is 1 ng/g.

The detection limit and detection time of the method used in this study were compared with other methods. The results are shown in Table 9. The comparison results show that the method used in this study is sensitive, rapid and simple.

## 4. Conclusions

In this study, a colloidal gold immunochromatographic test strip based on mAb was used to detect ofloxacin residues in meat products. The affinity of the prepared monoclonal antibody was 9.85 × 10^9^ L/mol, and the cross-reactivity with the other four analogs except MBF was less than 0.1%, with high affinity and good specificity. The IC_50_ of the established ic-ELISA method was 0.13 ng/mL, the detection limit was 0.033 ng/mL, and the recovery rate was 80%~120%. The visual detection line of the colloidal gold immunochromatographic test strip in three meat products was 1 ng/g. The above results indicate that the developed immunological detection method can effectively detect the residues of ofloxacin.

## Figures and Tables

**Table 1:** Serum test results after the fifth immunization.

Immunogen Mouse Number	OFL-BSA		
	1	2	3
OFL concentration (ppb)	50	50	50
Titer_1_ (OD_450_ nm)	2.113 ± 0.014	2.102 ± 0.11	1.479 ± 0.037
Titer_2_ (OD_450_ nm)	2.201 ± 0.020	1.985 ± 0.087	1.525 ± 0.033
Inhibition ratio_1_ (%)	24 ± 0.94	19 ± 2.16	15 ± 1.21
Inhibition ratio_2_ (%)	58 ± 3.77	50 ± 1.06	42 ± 1.95

Note. Titer_1_ (OD_450_ nm), Inhibition ratio_1_ (%): using a homologous coating antigen. Titer_2_ (OD_450_ nm), Inhibition ratio_2_ (%): using a heterologous coating antigen.

**Table 2:** Screening results of optimal blocking conditions and competition time of ic-ELISA.

	Blocking Condition					Competition Time/h			
	37 °C 1 h	37 °C 2 h	4 °C Overnight	4 °C 1 h	4 °C 2 h	0.125	0.25	0.5	1
A_max_	1.635	1.49	1.521	1.771	1.678	1.36	1.42	1.53	1.56
IC_50_	0.34	0.22	0.34	0.38	0.36	0.39	0.56	0.25	0.27
A_max_/IC_50_	4.81	6.77	4.27	4.66	4.66	3.49	2.54	6.12	5.78

**Table 3:** The screening results of the optimal NaCl content and pH in ic-ELISA.

	NaCl Content					pH				
	0%	0.5%	1%	1.5%	2%	5	6	7.4	8.6	9.6
A_max_	1.69	1.63	1.56	1.56	1.51	1.62	1.63	1.52	1.5	1.51
IC_50_	0.23	0.41	0.21	0.14	0.27	0.42	0.25	0.13	0.46	0.52
A_max_/IC_50_	7.35	3.98	7.43	11.14	5.59	3.86	6.52	11.69	3.26	2.9

**Table 4:** The cross-reactivity of OFL monoclonal antibody.

Chemical Compound	IC_50_ (ng/mL)	CR (%)
OFL	0.13	100
MBF	0.15	86.67
LOM	>500	<0.1
PEF	>500	<0.1
CIP	>500	<0.1
NOR	>500	<0.1

**Table 5:** The color of gold-labeled antibody solution at different pH values.

	Volume of 0.1 M K_2_CO_3_ Used for 1 mL of Colloidal Gold (μL)							
	0	4	6	8	12	14	16	20
Solution color	--+	--+	-++	-++	+++	+++	+++	+++

Note: “--+”: blue–violet; “-++”: purple; “+++”: red.

**Table 6:** The color of gold-labeled antibody solution at different antibody concentrations.

	The Amount of Antibody Used in 1 mL Colloidal Gold (μg)							
	4	6	8	10	12	14	16	18
Solution color	--+	-++	+++	+++	+++	+++	+++	+++

Note: “--+”: blue–violet; “-++”: purple; “+++”: red.

**Table 7:** The optimization for the concentrations of antigen and antibody on the colloidal gold test strip.

Antigen Concentration (mg/mL)	0.2			0.5			1		
Gold-labeled antibody (μL)	4	8	12	4	8	12	4	8	12
Negative T line gray value	243	478	668	507	899	1004	789	1328	1811
vLOD (ng/mL)	-	-	0.5	0.5	1	1	1	3	4

Note: “-” indicates that the T-line signal cannot be judged by the naked eye.

**Table 8:** Recovery rates of OFL (*n* = 3).

Samples	Added Standard Concentration (ng/g)	Recovery (%)	CV (%)
pork	0.00	-	-
	0.25	91.00 ± 1.00	2.56
	0.50	103.20 ± 6.40	6.12
fish	0.00	-	-
	0.25	82.00 ± 5.00	4.11
	0.50	99.60 ± 2.40	3.12
chicken	0.00	-	-
	0.25	86.00 ± 3.00	2.37
	0.50	104.40 ± 5.60	3.34

**Table 9:** Comparison of this method with other measurement methods.

Reference	Method	LOD (ng/mL)	Analysis Time (h)	Detection Object
This work	Colloidal gold immunochromatographic assay	1 (ng/g)	0.25	pork, fish, and chicken
	ELISA	0.033	5	
[5]	HPLC	20	1–2	influent, effluent, and surface waters
[6]	HPLC	3.6	1–2	human urine
[7]	HPLC-FLD	0.7	1–2	chicken meat
[8]	HPLC-MEPS-FLD	0.05 (ng/g)	1–2	milk
[9]	HPLC-MS/MS	0.005–0.123	1–2	surface water and hospital wastewater
[10]	HPLC-MS/MS	4.30 (ng/g)	1–2	milk
[11]	SPE- HPLC-MS/MS	0.0023–0.0107	1–2	environmental water
[12]	HPLC-MS/MS	0.1–3 (ng/g)	1–2	bean sprouts
[13]	SERS	42.6–49.1	1–2	aquatic samples
[14]	ELISA	25 (ng/g)	5	kidney, marine products, eggs, and muscle
[15]	ELISA	1.15	5	rana catesbeianus
[17]	immunochromatographic assay	3.5–8.9	0.25	milk
[1]	immunochromatographic assay	30	0.25	aquatic samples

## Data Availability

The original contributions presented in this study are included in the article/Supplementary Materials. Further inquiries can be directed to the corresponding author.

## Supplementary Materials

The following supporting information can be downloaded at https://www.mdpi.com/article/10.3390/foods13244137/s1, Table S1: Positive cell screening results of titer of No. 1 microplate; Table S2: Positive cell screening results of titer of No. 2 microplate; Table S3: Positive cell screening results of titer of No. 3 microplate; Table S4: Positive cell screening results of titer of No. 4 microplate; Table S5: Positive cell screening results of titer of No. 5 microplate; Table S6: Positive cell screening results of titer of No. 6 microplate; Table S7: Positive cell screening results of titer of No. 7 microplate; Table S8: Positive cell screening results of titer of No. 8 microplate; Table S9: Preliminary screening results of positive cells.
