# Supplementary material for: Development of a Colloidal Gold Immunochromatographic Assay Strip Using a Monoclonal Antibody for the Rapid Detection of Ofloxacin
Source: Foods. 2024 Dec 20;13(24):4137. doi: 10.3390/foods13244137 (PMC11675736; doi:10.3390/foods13244137)
Supplement: Supplementary file 1 [file foods-13-04137-s001.zip › foods-3315494-supplementary.pdf]

Supplementary Materials

Cell screening data are shown in Table S1 to Table S9:

Table S1. Positive cell screening results of titer of No.1 microplate.

	1	2	3	4	5	6	7	8	9	10	11	12
A	0.185	0.153	0.286	0.206	0.225	0.320	0.196	0.220	0.366	0.196	0.208	0.239
B	0.154	0.285	0.119	0.189	0.217	0.137	0.134	0.149	0.405	0.312	0.109	0.121
C	0.159	0.131	0.142	0.154	0.117	0.104	0.131	0.137	0.104	0.127	0.077	0.300
D	0.202	0.149	0.121	1.982	0.133	0.122	0.118	0.106	0.233	0.133	0.116	0.187
E	0.182	0.146	0.144	0.765	0.194	0.145	0.117	0.092	0.108	0.131	0.096	0.108
F	0.221	0.126	0.149	0.160	0.151	0.169	0.133	0.151	0.111	0.169	0.142	0.137
G	0.157	0.115	0.123	0.095	0.161	0.120	0.128	0.121	0.117	0.109	0.119	0.169
H	0.214	0.157	0.129	0.153	0.205	0.162	0.192	0.196	0.205	0.210	0.209	0.376

Table S2. Positive cell screening results of titer of No.2 microplate.

	1	2	3	4	5	6	7	8	9	10	11	12
A	0.200	0.161	0.250	0.186	0.204	0.170	0.171	0.194	0.156	0.165	0.208	0.208
B	0.246	0.128	0.264	0.158	0.170	0.200	0.117	0.169	0.115	0.125	0.184	0.164
C	0.280	0.143	0.152	0.112	0.118	0.209	0.132	0.146	0.278	0.111	0.132	0.151
D	0.165	0.193	0.143	0.114	0.133	0.218	0.117	0.119	0.097	0.091	2.116	0.124
E	0.183	0.120	0.108	0.088	0.120	0.146	0.090	0.106	0.093	0.087	0.097	0.130
F	0.140	0.095	0.130	0.094	0.109	2.458	0.111	0.111	0.091	0.078	0.132	0.122
G	0.168	0.141	0.146	0.135	0.121	0.145	0.095	0.094	0.118	0.110	0.119	0.131
H	0.201	0.194	0.164	0.208	0.219	0.189	0.205	0.131	0.157	0.161	0.168	0.239

Table S3. Positive cell screening results of titer of No.3 microplate.

	1	2	3	4	5	6	7	8	9	10	11	12
A	0.330	0.204	1.192	0.177	0.269	0.214	0.253	0.408	0.238	0.261	0.277	0.299
B	0.430	0.186	0.190	0.350	0.194	0.144	0.474	0.234	0.145	0.196	0.272	0.262
C	0.209	0.123	0.204	0.196	0.170	0.279	0.306	0.187	0.141	0.239	0.245	0.193
D	0.149	0.109	0.206	0.236	0.133	0.152	0.229	0.119	0.097	0.116	0.234	0.147
E	0.150	0.100	0.243	0.137	0.139	0.161	0.109	0.117	0.079	0.087	0.132	0.129
F	0.140	0.095	0.116	0.128	0.108	0.099	0.122	0.111	0.123	0.088	0.144	0.239
G	0.314	0.190	0.241	0.193	0.181	0.202	0.155	0.163	0.117	0.141	0.197	0.156
H	0.210	0.159	0.149	0.133	0.147	0.152	0.124	0.247	0.169	0.153	0.178	0.239

Table S4. Positive cell screening results of titer of No.4 microplate.

	1	2	3	4	5	6	7	8	9	10	11	12
A	0.191	0.317	0.189	0.285	1.219	0.204	0.318	0.234	0.200	0.295	0.184	0.160
B	0.271	0.186	0.205	0.176	0.268	0.282	0.146	0.235	0.170	0.214	0.174	0.285
C	0.279	0.291	0.187	0.354	0.322	0.249	0.165	0.238	0.246	0.258	0.218	0.164
D	0.199	0.135	0.251	0.150	0.146	0.152	0.116	0.169	0.141	0.116	0.125	0.155
E	0.318	0.171	0.173	0.137	0.231	0.176	0.278	0.191	0.123	0.087	0.092	0.128
F	0.266	0.237	0.178	0.111	0.174	0.157	0.139	0.229	0.151	0.089	0.120	0.184
G	0.300	0.190	0.135	0.154	0.147	0.132	0.142	0.117	0.094	0.120	0.119	0.146
H	0.337	0.387	0.292	0.231	0.265	0.232	0.236	0.301	0.124	0.462	0.178	0.220

Table S5. Positive cell screening results of titer of No.5 microplate.

	1	2	3	4	5	6	7	8	9	10	11	12
A	0.242	1.214	0.198	0.218	0.271	0.337	0.332	0.429	0.564	0.278	0.333	0.396
B	0.180	0.151	0.246	0.175	0.147	0.247	0.180	0.293	0.224	0.352	0.285	0.403
C	0.195	0.127	0.137	0.166	0.157	0.176	0.155	0.239	0.163	0.128	0.117	0.211
D	0.248	0.136	0.197	0.163	0.120	0.202	0.151	0.150	0.140	0.161	0.158	0.137
E	0.214	0.172	0.154	0.162	0.112	0.137	0.198	0.134	0.124	0.088	0.094	0.166
F	0.193	0.134	0.158	0.139	0.143	0.143	0.125	0.127	0.151	0.113	0.120	0.162
G	0.231	0.189	0.218	0.279	0.148	0.157	0.170	0.233	0.147	0.141	0.157	0.189
H	0.274	0.259	0.249	0.386	0.188	0.184	0.219	0.537	0.207	0.204	0.199	0.268

Table S6. Positive cell screening results of titer of No.6 microplate.

	1	2	3	4	5	6	7	8	9	10	11	12
A	0.243	0.227	0.443	0.355	0.297	0.311	0.526	0.356	0.318	0.372	0.332	0.395
B	0.171	0.128	0.233	0.166	0.145	0.226	0.226	0.193	0.263	0.229	0.224	0.305
C	0.185	0.194	0.157	0.133	0.141	0.176	0.147	0.159	0.115	0.310	0.178	0.195
D	0.207	0.136	0.206	0.115	0.137	0.121	0.136	0.114	0.110	0.140	0.130	0.161
E	0.245	0.134	0.125	0.115	0.127	0.136	0.130	0.144	0.123	0.113	0.141	0.192
F	0.217	0.144	0.148	0.153	0.143	0.142	0.125	0.127	0.111	0.099	0.136	0.162
G	0.220	0.191	0.162	0.193	0.143	0.155	0.150	0.156	0.142	0.138	0.155	0.222
H	0.253	0.228	0.203	0.254	0.190	0.194	0.168	0.177	0.165	0.186	0.198	0.249

Table S7. Positive cell screening results of titer of No.7 microplate.

	1	2	3	4	5	6	7	8	9	10	11	12
A	0.308	0.238	0.319	0.129	0.185	0.151	0.205	0.189	0.207	0.200	0.212	0.196
B	0.246	0.236	0.154	0.106	0.196	0.092	0.179	0.171	0.102	0.182	0.112	0.181
C	0.324	0.241	0.325	0.083	0.121	0.112	0.061	0.074	0.067	0.105	0.156	0.175
D	0.394	0.175	0.200	0.172	0.085	0.134	0.063	0.106	0.174	0.104	0.111	0.200
E	0.221	0.153	0.090	0.176	0.077	0.133	0.053	0.077	0.057	0.058	0.166	0.189
F	0.264	0.198	0.118	0.094	0.121	0.115	0.132	0.111	0.139	0.106	0.159	0.167
G	0.201	0.295	0.110	0.171	0.109	0.154	0.107	0.125	0.102	0.167	0.127	0.183
H	0.318	0.180	0.281	0.167	0.152	0.321	0.289	0.226	0.220	0.227	0.269	0.248

Table S8. Positive cell screening results of titer of No.8 microplate.

	1	2	3	4	5	6	7	8	9	10	11	12
A	0.195	0.164	0.153	0.150	0.224	0.150	0.121	0.229	0.161	0.137	0.177	0.130
B	0.235	0.148	0.119	0.121	0.107	0.232	0.092	0.106	1.087	0.100	0.124	0.121
C	0.226	0.154	0.242	0.218	0.142	0.085	0.112	0.413	0.092	0.080	0.102	0.126
D	0.189	0.149	0.102	0.102	0.094	0.169	0.064	0.078	0.094	0.094	0.096	0.125
E	0.197	0.175	0.133	0.121	0.114	0.117	0.082	0.109	0.067	0.082	0.096	0.162
F	0.207	0.184	0.204	0.161	0.169	0.156	0.132	0.151	0.092	0.096	0.095	0.166
G	0.220	0.191	0.162	0.193	0.143	0.155	0.150	0.156	0.142	0.138	0.155	0.222
H	0.253	0.228	0.203	0.254	0.190	0.194	0.168	0.177	0.165	0.186	0.198	0.249

Table S9. Preliminary screening results of positive cells.

Cell Number	OFL concentration (0 ppb)	OFL concentration (25 ppb)		OFL concentration (50 ppb)	
	Absorbance ($OD_{450\ nm}$)	Absorbance ($OD_{450\ nm}$)	Inhibition ratio	Absorbance ($OD_{450\ nm}$)	Inhibition ratio
1D4	1.236	0.064	95%	0.042	97%
2D11	1.198	0.059	95%	0.040	97%
4A5	1.257	0.054	97%	0.036	98%
5A2	1.191	0.794	33%	0.269	77%
8B9	1.137	0.073	95%	0.058	95%
